# Overview of Optical and Electrochemical Alkaline Phosphatase (ALP) Biosensors: Recent Approaches in Cells Culture Techniques

**DOI:** 10.3390/bios9030102

**Published:** 2019-08-23

**Authors:** Thanih Balbaied, Eric Moore

**Affiliations:** University College Cork, Sensing & Separation Group, School of Chemistry and life Science Interface, Tyndall National Institute, T12R5CP Cork, Ireland

**Keywords:** alkaline phosphatase (ALP), optical biosensors, electrochemical biosensors, nanomaterials, microarrays technology, Lab-on-chip technology

## Abstract

Alkaline phosphatase (ALP), which catalyzes the dephosphorylation process of proteins, nucleic acids, and small molecules, can be found in a variety of tissues (intestine, liver, bone, kidney, and placenta) of almost all living organisms. This enzyme has been extensively used as a biomarker in enzyme immunoassays and molecular biology. ALP is also one of the most commonly assayed enzymes in routine clinical practice. Due to its close relation to a variety of pathological processes, ALP’s abnormal level is an important diagnostic biomarker of many human diseases, such as liver dysfunction, bone diseases, kidney acute injury, and cancer. Therefore, the development of convenient and reliable assay methods for monitoring ALP activity/level is extremely important and valuable, not only for clinical diagnoses but also in the area of biomedical research. This paper comprehensively reviews the strategies of optical and electrochemical detection of ALP and discusses the electrochemical techniques that have been addressed to make them suitable for ALP analysis in cell culture.

## 1. Introduction

The detection of alkaline phosphatase (ALP) was commenced in the late 19th century, and was recognized as an enzyme family after Robison’s contributions in 1932 [1]. McComb et al. (1979) summarized several attempts by scientists for the detection of ALP and displayed several topics that are of significance for multidisciplinary researchers [1]. The primary importance of detecting ALP is to identify the possibility of diseases and carry out immediate preventive or treatment operations [2]. In point of care applications, ALP is known to be measured in routine blood tests with high levels of serum considered as indications of bone disease, liver disease, or bile duct obstruction and recently appears to be a significant independent prognostic biomarker for indicating cancers [3,4,5,6,7,8,9,10,11,12]. ALP levels have various reference ranges depending on age, gender and patient history. ALP can be defined as an enzyme that liberates phosphate under alkaline conditions and is made in the liver, bone, and other tissues [13].

**Figure 1:**
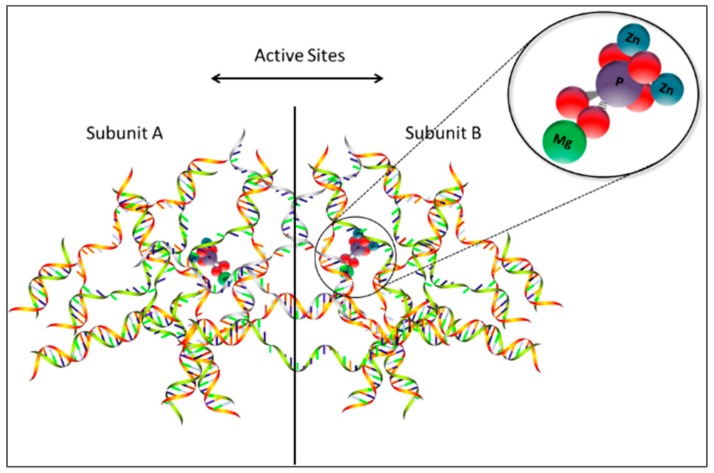
Illustrates the 3D structure of ALP shaped in two monomers (A and B). The central core of ALP (active sites) is formed by the link of the two monomers. The metals position in the edge of both monomers (red). In the licorice representation, the inorganic phosphate and metal ions are presented [16].

The X-ray crystallography technique characterizes ALP by obtaining a three-dimensional structure of the enzyme under study through diffracting its crystallized form [14]. Fersht et al. discusses how the three-dimensional structure is crucial in determining the functionality of the molecules. On the other hand, nuclear magnetic resonance technique uses energy transitions through a range of wavelengths. This technique characterizes enzymes by making a difference in the spectrum which appears in the strength of the electromagnetic field around the nucleus [14]. The approach thus provides an extended shift of efficiency allowing the determination of which type of atom or particles about their specific environment, thus, respective characteristics can be derived. Figure 1 illustrates that ALP is a homodimeric enzyme shaped in two monomers (A and B) where the central core of ALP (active sites) is formed by the link of the two monomers. The author elaborates that this property allows it to reach a stable heating capacity with a maximum capacity at very high pH [14]. Similarly, an active site found on the enzyme where one magnesium and two zinc ions located has the function of acting as a catalyst site. This occurs when mg^2+^ and zn^2+^ activate and inhibit, respectively, protein tyrosine phosphatase 1B (PTP1B). Millan describes the occurrence as molecular dynamics simulations, metadynamics, and quantum chemical calculations in combination with experimental investigations demonstrate that mg^2+^ and zn^2+^ compete for the same binding site in the active site only in the closed conformation of the enzyme in its phosphorylated state [15]. At this point, the cations have different effects on hydrolysis resulting in a difference in the establishment of the structural enzymology PTP1B.

Details of ALP isoenzymes and their test regarding their physical and chemical properties are discussed below. Furthermore, we have discussed some point taken into account the methods of discriminating between those isoenzymes.

## 2. ALP Isoenzyme and Tests

**Figure 2:**
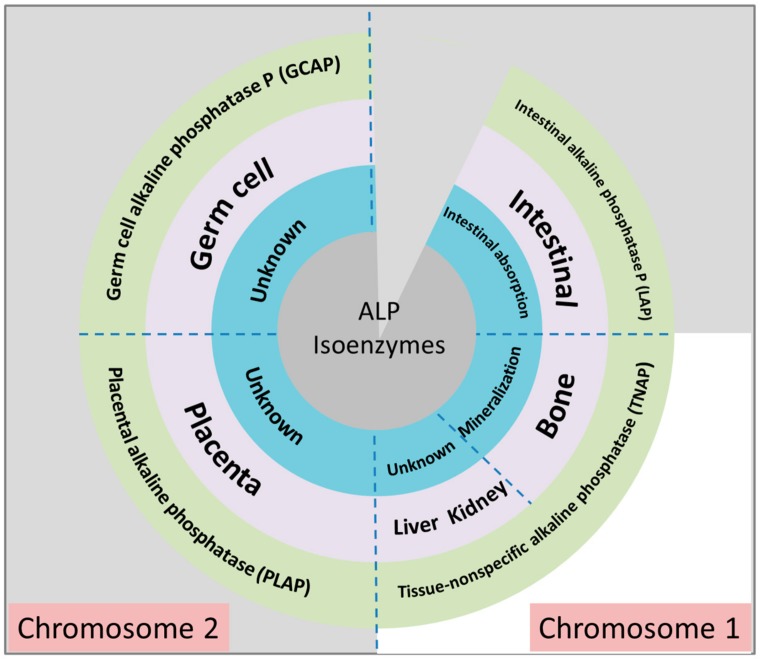
ALP isoenzyme with common names, place function limitation [19].

Mammalian ALPs are present as different isoenzymes. Figure 2 shows the main four isoenzymes of ALP, which are germ cell alkaline phosphatase (GCAP), intestinal alkaline phosphatase (IAP), placental alkaline phosphatase (PLAP) and tissue-nonspecific alkaline phosphatase (TNAP). GCAP, IAP and PALP, are located in chromosome 2, whereas TNAP is located in chromosome 1—all of which their precise physiological and neoplastic functions are unknown. However, TNAP was found to be responsible for calcification in bone and concern regarding regulating the secretory activities in liver, but as mentioned earlier their main function still unidentified. ALP isoenzyme can be referred to as a biomarker for cancer before a tumor is formed. Fishman (1980) contends that PLAP is sometimes found in individuals with ulcerative colitis or polyposis of the colon, and it is through this that they enhance the capability of acquiring cancer [17]. Bukowczan et al. also examined that PLAP has a relationship with different tumors such as renal cell carcinoma (RCC) [18].

Some physical and chemical properties are considered when discriminating between isoenzymes. Webster points out that the concentration of cysteine and histidine which inhibits alkaline phosphatase activity and the phosphotransferase activity of different isoenzyme preparations are similar [13]. Other physical and chemical properties include heat stability at 56°, electrophoretic mobility, and the concentrations of Zn ions, l-phenylalanine, and l-tryptophan required to inhibit enzyme activity by 50% are different. This variety was exploited to discriminate ALP isoenzyme. Some traditional methods depend on deactivating ALP, such as using selective inhibitors (e.g., l-phenylalanine, l-homoarginine, levamisole) or heat treatment. The classic approach is to heat the serum of ALP up to 56 °C for 10 min to distinguish between liver and bone ALP, for example. Whereas the other methods such as electrophoretic, isoelectric focusing and lectin-based rely on sieving media or non-sieving media. These methods use polyacrylamide, agarose gel, or the wheat-germ lectin for limiting the mobility of ALP isonenzymes in buffers. Moreover, separation-based methods are conducted to distinguish ALP isoenzymes, and are occasionally coupled with other techniques to give quantity of ALP. For example, chromatography methods such as affinity chromatography [20] have been applied to distinguish ALP isoenzymes and are occasionally associated with solid-phase immunoassay to give data of ALP levels. This method is still insufficient, although optimized by using wheat germ agglutinin (WGA) conjugate to the silica particle [21]. Liquid chromatography is applied instead with different anion exchange [22]. Per et al. used weak anion exchange to determine and separate ALP isoenzyme [23]. This assay improves sensitivity and selectivity over electrophoresis methods. However, the temperature for the analytical column must be strictly controlled, otherwise retention times and peak heights will be affected. These separation methods however, although useful as a research tool, have limited applications in the routine clinical laboratory.

Due to the complicated nature of ALP in terms of its physical and chemical properties and the fact that it has various levels between people and iosenzymes that indicate related diseases, particular expression systems are required to express ALP and makes it detectable in vitro assays, which will be expanded upon in the next section.

## 3. ALP Secretion System

**Figure 3:**
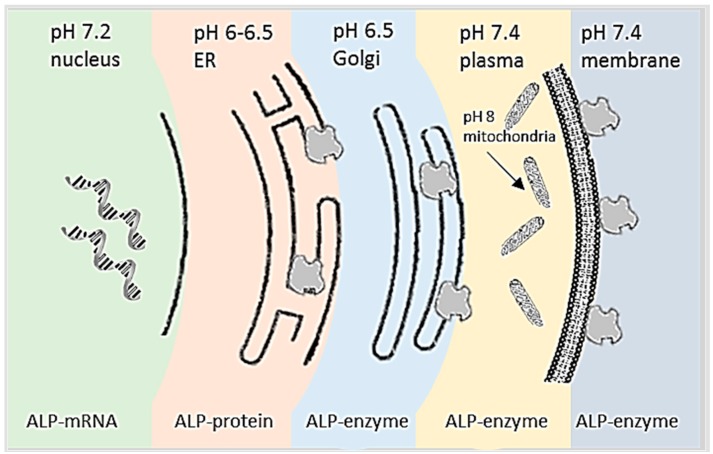
ALP produced in each stage in cells [26].

There are many expression systems for proteins including mammalian cell, bacteria and yeast. Among them, mammalian cells help in the folding of proteins, post-translational modifications and product assembly, which all are important in harnessing mammalian cells for protein production [24]. In addition, mammalian cells have proved to be well suited with an efficient expression system that leads to good productivity due to containing glycosylation, which is required for secretion and stability. However, a mammalian cell is more complex and costlier than other expression systems such as bacteria and yeast. ALP is located in the membrane of mammalian cells and is found to be in periplasmic space of *E. coli* bacteria [2]. Figure 3 shows that ALP is made in the nucleus. Then formed to protein in endoplasmic reticulum (ER) and in Golgi body formed as an iosenzymes. These processes are all under natural pH. ALP is then bonded to a cell membrane where the pH is 7.5. This pH does not allow ALP to induce unless the environment surrounding it rises to an alkaline pH. For example, during cell metabolism or necrosis inflammation where HCO^3-^ raises causes an increment of alkalinity in the cell membrane, thus activating ALP [25].

The technique of cell culture is efficient to study the physiological effect of ALP in vitro assays and develop cancer research. Cell culture is the technique of extracting cells from a living organism and growing them in an artificial environment producing cells lines simulating cancer in vivo [27]. The number of cell lines are ever increasing and this has attracted the need for finding the right model for cancer. For example, A549 cell lines investigated many biomarkers in lung cancer includes C4b-binding protein [28], micoRNAs [29], volatile biomarkers for apoptosis and necrosis [30] and ALP biomarker—which was significant in modulating A549 cell phenotype [31]. MCF-7 cell line was developed about 48 years ago with about 25,000 published reports, making it the most published cell line after the HeLa cell line, which has about 80,000 publications. MCF-7 studied many biomarkers such as P glycoprotein [32], doxorubicin-resistant [33] and 52 K glycoprotein [34] ALP [35]. HT-29 cell lines can express brush border linked to hydrolases which are common in the small intestines, though they have a lower enzymatic activity than that of in vivo cells. Ht-29 received a lot of interested in food digestion and bioavailability [36,37,38], the transport of drugs and food, immune response, adhesion and invasion of microorganisms [39] and diagnosing ALP as biomarkers in each stage of colon cancer [12] as well as studying the regulation of ALP [40].

There are various ions across lipid membranes in cells where ALP was found to be conducted due to the membrane permeability for K^+^ [41]. Along similar lines, Gerlach et al. argued that the membrane conductance of ALP is a calcium-dependent potassium channel (hIK1) [42]. Another study by Bo Yang et al. suggested that the calcium activated chloride channel A1 (CLCA1) may regulate the transition from proliferation to differentiation in colon cell lines; Caco-2 cells [43]. Another study found that ALP affected the concentration of calcium ion [44]. Patch-clamp and RT-PCR approaches help understand the functional and molecular expression of voltage-operated calcium channels [45]. As such, the researchers investigated whether voltage-operated calcium channels play a role in osteogenic differentiation of human bone marrows. Results revealed that mRNA for pore-forming 1C and 1G subunits of T-type and L-type Ca^2+^ channels were found to be present in comparable amounts in cells cultured in maintenance medium. The techniques were also found to be effective in detecting progressive bone mineralization of increased activity of ALP. Macrae et al. explored techniques for using synthetic derivative gramicidin A for the ion-channel forming peptide [46]. The device was developed to report variations in the local environment to external stimuli. The results revealed that gramicidin A ion channels give substantial detection for changes in ALP concentrations and environmental alterations.

A number of approaches have been extensively developed to understand the gene expression of ALP in vitro diagnostic tests where the reason for causing diseases as well as tracking the effectiveness of pharmaceuticals was unveiled. Therefore, accurate quantification of ALP level provides a signature of ALP function and gives a better understanding about the prognostic level of ALP in different cancers.

## 4. Traditional ALP Methods

There are three main traditional methods that have been adopted to achieve better quantification of ALP level. Firstly, the fluorescence-based methods include flow cytometry, histochemical and immunohistochemical. The sample precipitate with substrates such as enzyme-labeled flouresence-97 (ELF-97) phosphatase, naphthol phosphate, and menadiol diphosphate coupled with some of salts and dyes such as azo dye, diazonium salts and tetrazolium salts so as to produce insoluble colored products detected by fluorescence. These methods allow high sensitivity and fast detection. However, their expensive instrumentations hinder their convenient use alongside the need for highly skilled personnel [15,47,48].

Secondly, mRNA-based methods; northern blot [49] and a reverse transcriptase-polymerase chain reaction (RT-PCR) [50] can detect real-time ALP level. The former method is an old and classic method used to detect a specific isoform of ALP based on the level of its mRNA [49]. The RT-PCR approach is also based on RNA expression, but it combines with a single nucleotide primer extension assay to discriminate ALP isoenzymes [49].

Thirdly, immunoreaction-based method including western blot, radioimmunoassay (RIA), and enzyme-linked immune-sorbent assay (ELISA) have been conducted to gain more selectivity and sensitivity [51]. Western blot involves an electrophoretic sieving, which allows ALP separated by size and then the results transferred to a membrane producing bands. The membrane is incubated with labels of specific antibodies. This method, although sensitive, is time-consuming and has a high demand in terms of experience of the experimenter. Additionally, it requires multiple optimizations of the experimental conditions. In RIA, polyclonal are optimized to monoclonal antibodies to eliminate the cross reactivity. Thus, RIA is considered a method valid to detect ALP. RIA requires radioactive isotopes of iodine as an indicator. In such assay, a certain bead such as polystyrene coated with polyclonal rabbit anti-ALP are incubated with test samples and iodinated by chloramine—a radioactive compound—then, the radioactivity is counted by a gamma counter [52]. Although RIA is a sensitive method, it requires frequent preparation of radioactive antibodies as well as exposure to radiation hazards. Additionally, it require multiple steps for handle, storage, and disposal of radioactive materials.

One solution that succeeded to provide a similar sensitivity based solid phase and with no radiohazard is ELISA. In this technique, an enzyme conjugate and monoclonal antibodies are used. Colorimetric assay is then used in the presence of an appropriate chromophore (e.g., p-nitrophenyl phosphate) [53]. Although simple, this technique is a lab-based application [54]. In care-of-point application, ELISA based antibodies may cause false results [55]. For example, the human body can continue producing antibodies even though the person may have had the disease earlier and has recovered. In addition, some people are poor producers of an antibody or have some interfering substance in their blood, thus the amount of antibody may be too low to measure accurately or may go undetected. Cross reactivity may occur as a result of unrelated antibody reacts with the antigen non-specifically, thus bringing about positive signals for false reaction [56]. Another limitation with monoclonal antibodies is that immunoassays cannot identify the isoenzyme ALP [57,58].

Traditional methods mentioned earlier have some advantages, however; they are affected by requirements of multiple external controls for quantitative analysis as well as sequencing experiments to allow reliability. Additionally, a large sample volume, expensive instruments, and costly reagents are mostly needed. In addition, clinical ramifications need a simpler and more sensitive technique for testing of ALP expression.

Enabling high throughput technologies such as biosensors in point of care applications require some features such as portability, simplicity, and cost effective—all of which can be achieved through electrochemical methods. Literatures so far have developed methodologies for ALP detection in biological samples. For instance, Millan reviewed the use of conventional approaches in ALP analysis [15]. This paper is an updated version to solely cover for the available optical and electrochemical techniques for ALP detection. A brief outline of the recent advancements in optical detection strategies for ALP followed by electrochemical detection strategies are highlighted. More importantly, it was highlighted that recent technologies have been addressed to make each of the techniques suitable for ALP analysis in cell culture.

## 5. Optical Detection Techniques

The development of revolutionary technologies for imaging real-time events in living cells has been on the rise in the past decades. Today, enzymes have been put at work and optically acquired images have been significant in helping researchers to understand biological processes compared to abstract measurements [59]. Specifically, live-cell imaging with optical microscopy methods is powerful because they enable real-time detection of cellular processes [60]. Optical events are detected using bioluminescent and fluorescent probes that emit radiation at near-infrared or visible wavelengths, which are detected using optical cameras [60]. Optical methods have been used in detecting analytes because of their simple application. Cagnin et al. pointed out different optical techniques based on target labelling use radioisotopes, fluorophores, and UV-absorbing molecules. Fluorescence is used to elaborate molecular events results to light absorption when compounds such as polyaromatic hydrocarbons or heterocycles are used [61]. Upon being excited by light, these compounds can change their energy levels and decay from the excited state and in the process emit fluorescent light [61] In comparison, some optical methods such as non-linear optical sensing techniques and optical tomography and spectroscopy are based on label free detection where detection is achieved through nonpolar reactions, polar reactions, ionic reaction, hydrogen bonding, and covalent binding [62].

The common optical assays of ALP determination are most likely fluorescence, chemiluminescence, Raman spectroscopy, infrared spectra and colorimetric as well as surface plasmon resonance—all of which are expanded upon in the next section.

### 5.1. Fluorescence Methods

Fluorescence methods involve the use of fluorophore which is excited by energy light. Ejected electrons are transitioned within a molecule from ground state to an excited state. When the electrons relax, they fall to the lowest energy level emitting their energy into an emitted photon [61]. Fluorescence methods offer advantages such as being simple, specific and fairly sensitive and can detect low quantities in samples as well. Unfortunately, the fluorescence technique is limited to samples that are able to emit light [59,61]. Many strategies in fluorescence methods have been widely used in ALP detection involve real-time, label-free, affinity principle, and developing probes.

Deng et al. (2015) demonstrated a simple and effective radiometric fluorescent technique used in real-time detection of ALP based on stimulus-responsive coumarin@Terbium-guanine monophosphate nanoparticles [63]. The technique is real-time, sensitive, and robust as it has double signal response read-out with linear detection limits for ALP ranging from 0.025–0.2 U/mL. Qian et al. (2015) developed a label-free real-time fluorometric assay for highly sensitive ALP detection based on aggregation and disaggregation of CQDs using Cu^2+^ [64]. The presence of carboxyl groups on the CQDs enables aggregation due to fluorescence quenching by copper ions, with subsequent interaction among PPi, copper ions, and carboxyl initiating disaggregation inducing fluorescence emission. The new techniques can quantitatively detect ALP to high sensitivity levels of up to 1.1 U/L in human serum. Liu et al. (2016) designed a new fluorescence turn-on sensing technique for ALP activities based on the variations of graphene oxide (GO) affinity with double stranded DNA (dsDNA) and single-stranded DNA (ssDNA) coupled with λ exonuclease cleavage [65]. The new method exhibits high sensitivity to ALP with detection limits of 0.19 U/L—which is high enough in the practical determination of ALP in human serum. Qu et al. (2017) developed a single-step hydrothermal treatment of the *Sterculia lychnophora* seed with strong blue luminescence and water solubility [66]. The synthetic technique was found to be facile without post-treatment or complex techniques. The CDs-MnO_2_ nanocomposite as used as a new sensing probe for label-free and sensitive detection of ALP with its detection limit being as low as 0.4 U/L. The findings from these studies suggest that fluorescence assays can have an effect on vivo assays for real-time ALP detection. Chen et al. (2017) described a novel assay strategy for real-time detection of ALP in vivo based on the fluorescence quench-recovery technique at physiological pH using boron-doped GQDs as a fluorophore and ATP as a substrate [67]. The assay is able to discriminate ALP expression in cells up to very low concentrations of 10 ± 5 cells mL^−1^.

### 5.2. Chemiluminescence Methods

Chemiluminescence methods are based on the emission of light (luminescence) from a sample being investigated as a result of a chemical reaction. The luminescence reagent is continuously added to the sample and mixed with the column eluate in the mixer. The resulting luminescence from the chemical reaction is then measured using a photomultiplier tube when the luminescence raises to its highest intensity [68]. The advantages of chemiluminescence methods are limited to high sensitivity, broad dynamic range, and applicability over a broad spectral range, although during detection impurities can cause nonspecific background signals that degrade its sensitivity. The effectiveness of this essay may be seen in the strength of chemiluminescent. Sasamoto et al. (1995) studied the application of lucigenin as a chemiluminescent reagent in enzymatic activity assay for ALP detection [69]. The reagent reacted with hydrogen peroxide and phenacyl phosphate as a substrate for ALP detection. The phenacyl alcohol created an enzyme-catalyzed reaction with lucigenin giving luminescence with a detection limit of 3.4 × 10^−1^ mol and can be applied in enzyme immunoassays. Ximenes et al. (1999) developed hydrolytic action upon disodium (2-methyl-1-propenyl)phosphate (Na_2_MPP) to detect ALP activity [70]. The Na_2_MPP releases enol from isobutyraldehyde and is oxidized to HRP/H_2_O_2_ system producing chemiluminescence. The limit of detection (LOD) was 1.5 femtomole of ALP per assay, which is at the same range as other chemiluminescent methodologies. Kokado et al. (2002) developed a simple and sensitive chemiluminescent assay (CL) for ALP detection using dihydroxyacetone phosphate [71]. ALP hydrolyzation transformed new substrates to dihydroxyacetone, which reacted with lucigenin producing strong chemiluminescence. Under optimal conditions, the limits of detection were 3.8 × 10^19^ and 1.5 × 10^−18^ moles of ALP. When applied to enzyme immunoassay of 17β-oestradiol, the proposed CL method had a measurable range of 15–4000 pg/mL with four times more sensitivity compared to colorimetric assay for ALP when 4-nitrophenyl phosphate was used as the substrate. Overall, these strategies support the view that chemiluminescence methods can detect ALP in real-time. Meng et al. (2005) developed a new system for continuous and real-time monitoring of cross-talk between mesangial and macrophage cells in ex vivo and in vitro [72]. The technique provides simple and reliable tools for monitoring mesangial cells and macrophages which can be applied in the therapeutic management of infiltrating leukocytes and resident glomerular cells.

### 5.3. Raman Spectroscopy

Raman spectroscopy is based on the light scattering technique known as the Raman Effect. The Raman Effect relies on a fraction of small scattered radiation, which is different from the frequency of monochromatic incident radiation when incident radiation interacts with vibrating molecules. The scattered radiation gives information about the molecular structure of the sample. The laser line is used as the reference point with the peak measured as a shift from the laser line using vibrational energies associated with bonds in the molecules [73]. The advantage of Raman spectroscopy relates to its capacity to require very small samples and can be used with gases, liquids, or solids. Samples can also be analyzed directly in blisters, bags, or bottles with no vacuum requirements. However, the disadvantages include the inability to detect alloys or metals and that the Raman Effect is weak, resulting in low sensitivity. Other disadvantages include equipment cost depending on their applications. A recent study reported by Cottat et al. (2017) supported the hypothesis that Raman spectroscopy can detect ALP [74]. They examined variations between phosphorylated and unphosphorylated protein forms (e.g., Spleen Tyrosine Kinase (Syk)). Spectral similarities between dephosphorylated-Syk and unphosphorylated-Syk indicated that phosphorylated Syk was able to reverse its conformation after unphosphorylated due to phosphatase treatment. This study would seem to suggest that using advanced materials and the affinity approach can be used for avoiding low sensitivity. Yang et al. (2017) develop a new surface-enhance Raman spectroscopy (SERS) immunoassay to high selectivity and sensitivity of cancer markers such as AFP using enzyme-assisted Agdeposition on 4-mercaptobenzoic acid labeled gold nanoparticles (AuNPs) seeds [75]. The formed Au@Ag nanostructure had strong SERS signal detection for AFP ranging from 0.5 to 100 pg/mL with LOD of 0.081 pg/mL (3σ). Bozkurt et al. (2018) developed alternative methods using ALP activity for *E. coli* detection [76]. Three steps were using including modification of spherical magnetic gold coated core-shell nanoparticles and application of immunomagnetic separation to capture *E. coli* from solution. The ALP was immobilized on Au-NRs, and indirect detection of *E. coli* base on SERS done. Satisfactory limit of quantification (LOQ) (30 cfu/mL) and LOD (10 cfu/mL) were detected using sandwich immunoassay in less than 3 h.

### 5.4. Infrared Spectra Techniques

Infrared spectra techniques use the principle that molecules appear to absorb specific light frequencies characteristic with their molecular structure. The energies rely on the shape of molecular surfaces, mass corresponding to atoms, and associated vibronic coupling. Molecules, for example, are able to absorb energy present in the incident light and this results in either pronounced vibration or faster rotation [73]. The advantage of the infrared spectra method is that it does not destroy the sample and can thereby give qualitative and quantitative chemical data about the sample. The sample preparation process is simple with no specific requirements, and the spectra are very sensitive—even in small samples—in addition to being versatile in gas, liquid, solid, and semisolid detections. The disadvantage of the technique includes difficult handling procedures and sample maintenance, with the use of properly tuned and highly sensitive devices. Ren et al. (2015) demonstrated that inferred spectroscopy can detect phosphatase activities in situ; in osteoblast cells with the use of natural substrates without labeling [77]. The researchers recorded overall phosphatase activity in cells through a single step via substrate and protein concentration measurements. Specific activity in osteoblasts was 116 ± 13 nmol/min/mg for PPi to 56 ± 11 nmol/min/mg for AMP, to 79 ± 23 nmol/min/mg for beta-glycerophosphate and to 73 ± 15 nmol/min/mg for 1-alpha-D glucose phosphate. Furthermore, the absorption bands were recorded 1107 cm^−1^ for PPi, 977 cm^−1^ for AMP, 990 cm^−1^ for beta-glycerophosphate, and 990 cm^−1^ for 1-alpha-D glucose phosphate. It appears likely that the high standard deviation is due to protein concentration. Although the overlap was recorded in this assay, a definite need for a novel probe appears to be required. Li et al. (2017) reported a novel near infrared fluorescent probe based on hemicyanine dye in detecting endogenous ALP activity [78]. The new probe exhibited high sensitivity to ALP with 10-fold improvement when 2.0 U/mL ALP is added. Gao et al. (2018) designed and synthesized a near-infrared fluorescence probe to image and assess endogenous ALP changes in different tumor line cells [79]. The probe contained two parts: heptamethine cyanine as fluorescence modulator and phosphate monester as a response moiety for ALP enzymatic reaction. The technique showed high sensitivity to ALP detection in cancer cells. Wu et al. (2018) designed an ALP-activatable near-infrared photoacoustic probe [80]. Once the ALP is dephosphorylated, it self-assembles into nanostructures with enhanced photoacoustic signal for tumor imaging. The new device had 2.3-fold in detection limit at 4 h after 1P injection. Xu et al. (2019) made a successful synthesis of hemicyanine dye-mimic near-infrared fluorescent probe (NIR MTR) where they used 2-methylbenzo[d]- thiazole to replace 2,3,3-trimethyl-3H-indole [81]. The developed probe exhibited good ALP sensing performance showing detection limit of 0.042 U/L and linear range of 0–8 U/L.

### 5.5. Colorimetry Methods

Colorimetry methods are based on the Beer-Lambert’s law where light absorption transmitted through a medium is considered to be directly proportional to the concentration in the medium. In sample detection, a beam of light of a specific wavelength passes through a solution through several lenses that navigate the colored light to a measuring device. The device analyzes the color and compares it to the known standards and the microprocessor is used to calculate the percentage of transmittance or absorbance [82]. The advantages of the colorimetry technique include being fast, inexpensive, lightweight and portable, requiring a small sample size, minimal instrumentation and power, and the ability to customize array for specific analytes. However, some of the shortcomings include reproducibility of printing, reproducibility of imaging, difficulty of determining individual components of a mixture, and stability/shelf life. Chen et al. (2016) developed a low-cost, rapid, simple, and highly sensitive colorimetric technique using microfluidic paper-based analytical devices (μPADs) for monitoring serum ALP levels [83]. The coloration was recorded using Gel documentation systems. The LOD was noted to have a low detection limit of up to 0.78 U/L serum ALPs. Hu et al. (2017) developed a versatile technique for a selective and sensitive colorimetric assay for ALP activity based on absorption properties of Fe(II)-phenanthroline [84]. The technique exhibited good quantitative detection of ALP activity over a range of 0–220 mU mL^−1^ with a detection limit of 0.94 mU mL^−1^. It is likely that novel probes will improve colorimetric assays. Hu et al. (2017) proposed a practical and simple method for the selective and sensitive colorimetric assay of ALP activity [85]. The researchers used a water soluble and biologically conducive Cu(II)-phenanthroline complex as a probe. A two-step chromogenic reaction was used and was responsible for the turn-on spectral absorption in the visible region in addition to a distinct color change in the solution absorbed at the 424 nm band. Without the need for complex instruments and protocols, the new technique allowed for a sensitive readout of ALP activity where its linear range was wide at 0–200mU/mL, while the detection limit was down to 1.25mU/mL. Huang et al. (2018) developed a metal co-factor free deoxyribonucleic acid (DNA) and found that it displays H_2_O_2_ activity under mild conditions when acetic acid is used as an activator [86]. The new device demonstrated that in situ generations of peracetic acid results from guanine-rich oligonucleotides facilitation and subsequent tetramethylbenzidine (TMB) oxidation. The device had a detection limit of ALP in the linear range from 0.05 to 15 mU/mL, giving the LOD of 0.01 mU/mL. The new device can be used for colorimetric tests in analyzing real samples from human serum. Both Hu and Hu share LOD higher than Chen’ findings. These results would therefore appear to point out a definite need of improving more sensitive probes. Wu et al. (2019) have demonstrated that prussian blue nanocubes (PB NCs) can be used to make colorimetric probes for sensitive and selective monitoring of ascorbic acid and ALP activities [87]. The method is based on peroxidase activity of PB NCs which can be inhibited by ascorbic acid due to PB NCs being reduced to Prussian White. The method showed it can be used to detect ALP activity with a detection limit of as low as 0.23 U·L^−1^. Generally, optical methods show several advantages including being non-intrusive, presenting high accuracy, real-time detection, and being robust in ALP detection. However, they remain expensive, fragile, influenced by the environment, and are not readily portable.

### 5.6. Surface Plasmon Resonance

Surface plasmon resonance is a resonant oscillator based on electron conduction at the interface between positive and negative permittivity materials that are triggered by an incident light. The generated incident photons are absorbed at the metal layer surface by free electrons before being converted to surface plasmon waves when some conditions such as incidence angle, polarization, and wavelength have been achieved [88]. Researchers have further explored the properties of ALP from the surface plasmon resonance (SPR) perspective. For instance, Linder et al. (2016) explored the binding properties of human collagen and ALP of human bone through SPR analysis, and validated their research using blotting and electrophoresis [89]. Results revealed that bone ALP binds strongly to collagen in contrast to ALP detected in non-mineralizing tissue samples, and that binding affinity increased as the response rose. Sappia et al. (2017) researched SPR for the real time detection of ALP by using WGA-modified surfaces, where ALP absorbs and the signal increases [90]. Wang et al. (2018) created a probe using gold nanoflower in detecting cellular ALP and found that the ALP activity could be detected up to limits of about 0.03 μU L^−1^ [91]. In addition, the ALP activity of the mammalian cells was able to be attracted using the develop probe.

Nonetheless, despite clever and manipulating tactics of optical methods in ALP detection, the use of fluorophore can be affected by the biological events and cause low fluorescence intensity [29]. The fluorophore may lose its fluorescence during tissue fixation or subsequent processing. In addition, reagents should be fresh enough, placing samples in appropriate positions, and recording should be immediate—otherwise false values will be recorded as well as the simplicity of the colormetric may give insufficient resolutions. Moreover, experiments in vitro assays usually require multiple steps to prepare sample. Therefore, enabling electrochemical detection in vitro assays will minimize the steps of washing samples, centrifuge etc., thus limiting disturbing samples and obtaining appropriate results with less errors.

## 6. Electrochemistry Detection Techniques

Electrochemical methods give good grounds for expecting efficiency and sensitivity over optical methods. It was noted that optical devices are commonly used in laboratory settings due to their simplicity in microfluidic-detector interface. However, electrochemical detection offers better detection limits than optical devices for different biological analytes [92,93]. Also, it is easier to integrate electrochemical devices to microfluidic components within different chips in assay detection [94]. As a result, electrochemical methods offer real-time monitoring of biological occurrence and convert it into electronic signal. This type of signal can be easily integrated and observed in microdevices [95].

Electrochemical methods accomplish the measurement of ALP levels in vitro assays. The ALP level is determined according to different electrochemical signals including current, potential, conductivity and impedance. A certain reaction occurs in the presence of a particular substrate and produces electroactive properties such electron transfer, potential change, ionic species or current resistance. Thompson et al. (1991) compared the resolution of ALP resulting in amperometric detection over optical detection and found that amperometric had results 20 times better than that of optical [96]. The research of Thompson et al. has contributed to developing ALP electrochemical detection, which makes researchers to take advantage of Thompson’s contributions and apply different approaches such as using various substrates, self-assembled monolayers, immunoconjugates, and label free. In light of these considerations, it will be shown that several studies could detect secreted ALP in biological samples such as serum, cells, tissues and biopsy as well as by using different tactics for amplifying electrochemical signals. Electrochemical assays such as amperometric, potentiometric, and conductometric are common in detection ALP; therefore, they are further explained in the next paragraphs.

### 6.1. Amperometric

Amperometric techniques work by monitoring the change in a current once a constant potential has been applied. Potential changes are closely connected to enzymatic reduction and oxidation reactions inside the electrolyte solution within the working electrode [97]. In the process, the current can be compared to the electro-active specie concentration inside the sample. For instance, the presence of ALP works to hydrolyze o-phosphate at the p-aminophenyl phosphate (pAPP), where the non-electroactive substrate under the alkaline environment serves to generate an electroactive enzyme which contains redox cycling characteristics. Taking advantages of this principle allow for many studies to develop the detection of ALP in biological samples. Kelso et al. (2000) were most likely the first to detect secreted ALP in media using screen printed electrodes and 2-naphthyl phosphate [98]. They infected CHO cell lines to release ALP, which showed sufficient results that could be further optimized to increase sensitivity. On their print, a foot number of studies showing up developed amplifications. For example, the electrochemical signal of p-aminophenol can be amplified by bioelectrocatalysis using diaphorase and NADH, for instance. Ito et al. (2000) have used Tyrosinase with phenol phosphate to reduce the over-potential and obtained detectable results in the bovine serum. Although phenol phosphate has good stability, it needs two reactions to be detected [99]. Wang et al. (2009) have minimized the reaction in one single step, but they did not use any biological samples [100]. Shi-Ping et al. (2012) have used p-nitrophenol phosphate; the common optical substrate; and used ionic liquid to amplify the electrochemical signals [101]. They detected ALP in range of 1–100 UL^−1^ with good linearity. They did not use secreted ALP in their measurements. Xia et al. (2013) developed more sensitive electrochemical sensors for ALP detection using p-Aminophenol redox cycling [102]. The researchers compared the performance of various reductants in p-Aminophenol redox cycling using self-assembled monolayers that were on modified gold electrodes. For redox cycling, three reagents were found to be suitable in enhancing detection including cysteamine, tris(2-carboxyethyl) phosphine (TCEP), and nicotinamide adenine dinucleotide (NADH). The reaction rate in electrochemical detection of ALP decreases in the rate of cysteamine <TCEP <NADH. Some other methods used self-assembled monolayer (SAM) but without substrates. For example, Zhang et al. used a self-assembled monolayer technique where in the absence of ALP, they immobilized phosphorylated peptides (CP_P_Y) onto gold electrodes forming the negatively charged self-assembled monolayers (SAMs), and in the process it enabled access to positively charged [Ru(NH3)6]3+ probes onto the surface of the electrode. Once the electrode was incubated with ALP and CP_P_Y, the ALP removed the phosphate group in CP_P_Y. Although this trend uses cheap reagents, it allows for the indirect detection of ALP. Zhang et al. (2015) further developed a highly sensitive and facile homogenous electrochemical biosensor technique for ALP detection based on a single molecular exonuclease-assisted signal amplification [103]. The model was able to directly detect ALP levels up to 0.1 U/L, which is better than fluorescence methods and up to three times more sensitive than the immobilization-based electrochemical methods. These contributions aided to enable direct monitoring of ALP activity in living cells. Yildirim-Semerci et al. (2015) used an electrochemical assay for indicating the activities of cell-bound ALP using voltammetry on in-vitro cell culture [104]. The assay was based on p-aminophenol oxidation indication using square wave voltammetry, cell cultivation on gold microelectrodes on microplate wells, and catalytic hydrolysis of p-aminophenyl phosphate by ALP attached to cells. Detection of ALP activity was achieved through a signal increase associated with the number of cells and the rate of p-aminophenol formation rate. The obtained findings based on electrochemical activity assay were in line with calorimetric results obtained from p-nitrophenol formation rate. Porat-Ophir et al. (2015) have also reported the high effectiveness of the electrochemical technique in ALP detection using an integrated ’tissue on a chip’ model [105]. The model is based on detecting ALP activity using 1-naphthyl phosphate substrate from tissue samples placed on micro-electrochemical cells. Vernick et al. (2011) have compared healthy and unhealthy tissue using biopsies samples. Their investigation supported the fact that states that health tissues of intestinal exhibit higher activity of ALP than cancerous ones. Their direct electrochemical measurement assay of multiple samples uses a high capacity biosensing chamber which enables for the analysis of cell differentiation [106]. Ragones et al. (2015) presented a new electrochemical sensor based on an exceptional 3D architecture that allows for the direct measurement detection on close proximity or in contact with biological samples [107]. The chip was made of biocompatible substrates of electrochemical cells with a silver/silver chloride electrode and two gold electrodes (working and counter electrodes). The sensor had a stable signature and high detection response to ALP enzyme even in repeated tests. All the studies reviewed so far, however, try to avoid the disadvantages of amperometry assays.

### 6.2. Potentiometric

The potentiometric method works on a similar principle alike the amperometric measurement, but the difference is that there is no potential applied. Instead, the circuit remains open and the potential difference observed between the two electrodes is recorded [108]. During redox reactions, potentiometry is able to measure changes in cell potential in addition to monitoring the concentration of ion gradients via the ion-selective membranes. Moreover, potential changes on the working and reference electrodes are measured (commonly the Ag/AgCl electrodes are used) and this can be modified to improve the selectivity in sample detection through membranes. Keyes allow potentiometric detection of ALP in aqueous fluids. Katsu et al. (1996) constructed a hordenine sensitive membrane electrode and is used to detect ALP in blood serum [109]. Keyes allow for potentiometric detection of ALP in aqueous fluids. Katsu et al. (1996) constructed a hordenine sensitive membrane electrode and used it to detect ALP in blood serum [110]. The hordenine electrode was constructed using 2-fluoro-2’-nitrodiphenyl ether as a solvent mediator and sodium tetrakis [3,5-bis(2-methoxyhexafluoro-2-propyl)phenyl] borate was used as an ion-exchanger. The results confirmed that the device had high sensitivity to ALP compared to detection done using colorimetry and phenyl phosphate substrate. Many studies utilized the ability of ALP to catalyze monofluorophosphate hydrolysis alongside using fluoride ion-selective electrode to sense fluoride release to detect ALP. The amount of fluoride ion generated from the substrate in the course of the reaction is proportional to ALP activity. Koncki et al. (2005) developed a simple potentiometric assay in evaluating alkaline and acidic phosphatase activities [111]. Enzymatic catalyzation of monofluorophosphate was investigated as the primary focus of the assay. In the course of the hydrolysis, fluoride ions formed were detected using conventional fluoride ion-selective electrode based on a membrane made from lanthanum fluoride. Maximal sensitivity of acid phosphatase was observed at 10^−3^ M at pH 6.0; a pH of 4.8 was recommended to eliminate the effect of alkaline phosphatase. Koncki et al. (2006) developed a simple flow injection system for potentiometric detection of ALP activity. The researchers used monofluorophosphate as an ALP specific substrate [112]. The use of the substrate helped in improving the application of fluoride ion selective electrode in detecting enzyme-catalyzed reactions. Moreover, the low cost and chemical stability of monofluorophosphate makes it possible to use it as a substrate for the component carrier. Results show that the device allows for an inhibitive detection of vanadate and beryllium ions at ppb levels that have high selectivity, high throughput on the system, and a short time of analysis at nearly 8 samples per hour. Ogończyk et al. (2007) developed a flow injection system to detect ALP activity in human serum samples [113]. The researchers used an inexpensive and specific monofluorophosphate as an ALP substrate for their kinetic assay. The study also applied LaF_3_-crystalline membrane for biocatalytic hydrolysis of monofluorophosphate to enhance detection of fluorine ions on the ion-selective electrode. The optimized system showed high relativity and sensitivity to the short-time analysis of ALP of 5–6 samples per hour. The volume of the required serum was 0.05 mL and the system can detect ALP levels in human serum samples at pathological and physiological levels, and also in detecting the iso-enzymatic forms of ALP. Hassan et al. (2009) developed a new poly (vinyl chloride) matrix membrane detector responsive to 4-nitrophenylphosphate in assaying ALP and potentiometric assay [114]. The sensor was based on using an ion-association complex of 4-nitrophenylphosphate with a nickel (II)-bathophenanthroline cation as electrode active material, while the solvent mediator was nitrophenyloctyl ether. Results indicated that the sensor displays good stability and selectivity in detecting ALP and potentiometric assay of acid enzymes in patients suffering from prostate cancer, acute myelocytic leukemia, and alcoholic cirrhosis. For real-time ALP potentiometric methods, Kanno et al. (2016) described potentiometric bioimaging for enzyme activities based on a large scale integration-based electrochemical device of up to 400 sensors [115]. The potentiometric mode was used in the detection of ALP and glucose oxidase enzyme activities. The findings revealed that the enzyme activities were quantitatively detected in concentration ranges of 25–250 μg/mL for glucose oxidase and 0.10–5.0 ng/mL for ALP.

### 6.3. Conductometric

Conductometric methods work by measuring conductivity as a function of ionic species in a sample solution. The device contains two electrodes that are positioned to measure the conductivity of an electrolyte material that is adjacent to the surface of the electrode [97]. For instance, in the presence of electrolytes, ALP dissolves and in the process it induces catalytic reactions that are able to generate ionic species. This method does not involve a reference electrode, which makes it simpler than amperometric and potentiometric methods [116]. Guedri and Durrieu (2008) designed a conductometric biosensor to detect ALP activity in water using the microalgae *Chlorella vulgaris* [117]. The biosensor consisted of a microalgae bioreceptor and a transducer formed by platinum electrodes. The device had good repeatability measurements. Upadhyay and Verma (2015) developed a new and simple conductometric biosensor for indirectly determining phosphate ions in solution [118]. The biosensor was based on the inhibition of immobilized ALP in the presence of phosphate ions. Results indicated that the biosensor had a broad linear response (when compared to biosensors reported in past studies) for phosphate ion detection ranging from 0.5 to 5.0 mM with a correlation coefficient of R^2^ 995. The researchers also used different optimized parameters as a buffer at concentrations of 30 mM, pH 9.0, and substrate concentration of 1.0 mM for the device. Chouteau et al. (2004) developed a biosensor based on immobilized *Chlorella vulgaris* and tested it the using ALP analysis [119]. The sensor was used in detecting toxic compounds such as cadmium ions in the marine environment. The algae were immobilized in bovine serum albumin membranes and cross-connected with glutaraldehyde vapors. When compared to bioassays, Chouteau et al. noted that the results of conductometric biosensors using algae appeared more sensitive in detecting low-level cadmium ions. Besides, the ALP based biosensor is highly sensitive due to its specificity to toxic compounds. Compared to the use of biological systems, Berezhetskyyab et al. (2008) created an ALP based conductometric biosensor that contained enzyme membranes and interdigitated gold electrodes were used in assessing heavy-metal ions in water [120]. The analytes acted as enzyme inhibitors and the findings show that toxicity of different metals that were tested ranged in the order of Cd^2+^ > Co^2+^ > Zn^2+^ > Ni^2+^ > Pb^2+^. The limits of detection were about 40 ppm for Pb^+^, 5 ppm for Ni^2+^, 2 ppm for both Zn^2+^ and Co^2+^, and 0.5 ppm for Cd^2+^.

## 7. Other Detection Techniques

The subsequent techniques utilised their exclusive characteristics in detecting ALP either directly, investigating some of its properties, or applying it as a label. The utilized techniques are electrochemical impedance spectroscopy, quartz crystal microbalance, and field-effect transistor—all of which enable real-time detection.

### 7.1. Electrochemical Impedance Spectroscopy

Electrochemical impedance spectroscopy denotes the frequency-dependent resistance in terms of its relation with current flow in circuit elements (inductor, capacitor, resistor etc.) [121]. In this measurement type, it can either detect the capacitance at the electrolyte/electrode interface or it can detect stray capacitance between two opposite electrodes by responding to minute amplitude AC voltage. The impedance spectroscopy methods make it possible to monitor changes of mobile or linked charges on the volume around the interface regions. In 1996, Cai et al. purposed an impedance device based on a surface acoustic wave [122]. Their device allows conductivity of ALP measured in human serum samples where the higher ALP, the higher variations occur in the oscillation circuit. Lee et al. (2018) described a new impedimetric method of detecting ALP based on Cu^2+^ mediated oxidation of ascorbic acid on specific DNA probe-modified electrodes [123]. Researchers used PPi able to complex with Cu^2+^ as the ALP substrate enzyme. Free copper ions were bound to poly-thymine DNA probe attached to the surface and reduced forming copper nanoparticles through concomitant oxidation of ascorbic acid. The electrode surface accumulates the oxidation products and this increases electron transfer resistance as it interrupts the flow of electrons on the electrode. In contrast, the absence of ALP means that the PPi remains constant to suitably capture Cu^2+^, preventing the oxidation of ascorbic acid and continued the increase of electron transfer resistance. The detection rate was higher than results from electrochemical impedance spectroscopy with ALP detection of 6.5 pM (7.2 U/L) and also displays excellent selectivity.

The affinity approach was used in this technique. For example, Lucarelli et al. (2005) used an enzyme-linked electrochemical genosensor for ALP detection [124]. They describe the process of optimizing the performance of an enzyme-linked electrochemical genosensor created using a disposable oligonucleotide screen printed on gold electrodes. Researchers performed a qualitative analysis on the thiol-tethered probe using faradic impedance spectroscopy. Obtained impedance spectra revealed that thiol moiety contributed to unambiguous immobilization of oligonucleotide probe. Moreover, both hybridization efficiencies and probe surface densities were quantified via chronocoulometric measurements. Moreover, the researchers performed electrochemical transduction of the hybridization process via faradic impedance spectroscopy. The detection limits from the device were 1.2 pmol/L, demonstrating the usefulness of impedimetric genosensor in detecting ALP in samples. Ferancova et al. (2017) investigated the immunoassay events using three different electroactive substrates 1-naphthyl phosphate, 2-phospho-1-ascorbic acid, and hydroquinone diphosphate (HQ) as an opposite of chromogenic substrates in immunoassays [125]. Their result show that the substrates are significantly affected by blocking agents, but HQ is not affected. Another one was the adsorption approach, which is similar to immobilization. Shrikrishnan et al. (2012) detected ALP on a self-assembled monolayer modified gold electrode [126]. Their results show a decrease in impedance resolution when the protein of ALP adsorbed on to the monolayer due to the increment of ionic charges resulting in their reactions. Their study allows for a simple kinetic investigation of ALP comparable to ELISA. Advanced materials were used due to their affinity properties. Mintz et al. (2018) undertook an electrochemical impedance study to detect ALP in a phosphate buffer saline (PBS) solution using anti-ALP functionalized silicon nanowires [127]. The nanowire surface was modified by immobilizing the anti-ALP. The results indicated that the device was highly efficient in detecting low levels of ALP in the PBS solution ranging from 0.03 to 0.3 U/L with high selectivity due to antigen-antibody interactions. Kaatz et al. (2012) developed a device based on the impedimetric detection of enzymatic signals on the electrode surface [128]. The study demonstrated a technique which gives the needed detection levels at significantly reduced levels of enzyme reaction times and also shows the needed detection can discriminate samples. The study opens up to the potential of relevant and rapid multiparameter impedimetric ALP assays in the future.

### 7.2. Quartz Crystal Microbalance

Quartz crystal microbalance can detect change in a tiny mass as it monitors the frequency of oscillating shifts in crystals as a result of changes in pressure on the crystal surface resulting from mass loading. As the mass loading increases on the sensitive surface, there is a reduction in oscillation frequency [129]. ALP activities were analysed using quartz crystal microbalance. The approach is meant to increase the existing biotechnological application of microchips, protein arrays, and biosensors that are focused on kinetic immobilization of enzymes. The unique features of ease of genetic manipulation, self-assembly, and recognition make binding affinity a reliable molecular tool for site-specific enzyme immobilization Ebersole and Ward (1988) described amplified mass immunosorbent assay using crystal microbalance in detecting ALP and human chorionic gonadotropin [130]. The results indicated a high detection rate of the ALP and hormones on the quartz-crystal microbalance surface. The technique contributed to enzymatic amplification detection with significant enhancement of the detected limits. Kacar et al. (2009) demonstrated the use of the gold-binding peptide as a molecular linker immobilized on a gold substrate and genetically linked to alkaline phosphatase [131]. The enzymes were expressed in *E. coli* cells and gold bindings peptides focused on the N-terminus of alkaline phosphatase. The device demonstrated self-immobilization of the bi-functional enzyme on a micro-pattern substrate, while genetically linked end showed high enzymatic activity per area. The findings show a promising use of inorganic binding peptides as sites for specific molecular linkage for enzyme immobilization with retained activities. Thammasittirong et al. (2011) determined quantitative binding analysis of ALP immobilized on a gold electrode using a quartz crystal microbalance, which showed decrement in frequency at binding of Bacillus thuringiensis toxin (Cry4Ba) as a result of the mass raise [132].

### 7.3. Field-Effect Transistor

Field-effect transistor methods are designed around semiconductor material that consist of metal-oxide semiconductor structures. Any changes in the metal potential results in the induction of the electric field triggering the band bending of the semiconductor channel appropriately [133]. The process then results in changes to the channel carrier concentration including inversion, depletion, or accumulation. Most studies used field-effect transistor in terms of ALP applications where applying ALP as a label is fundamental of other methods. For example, Jang et al. (2015) demonstrated a new immunoassay ELISA based on an electrochemical method for optical and electrical signaling. The process was achieved by combining an ion-sensitive field effect with Enzyme-Linked immunoassay [134]. The device was then set to harness a catalytic reaction of alkaline phosphatase which precipitated silver particles. Small signals that ranged from 1 pg/mL to 10 ng/mL were greatly amplified with ALP labeled despite the buffer conditions. Results revealed that the developed sensor platform outperformed the sensing capacity of conventional ELISA, which is considered to have a LOD of about 1 ng/mL. Obtained findings were also compared with the results of horseradish peroxidase, which is commonly applied in the optical analysis of ELISA. The findings showed that the immunoassay device based on the ion-sensitive field-effect transistor (ISFET) based on portable sensors and can be used as a point of care tool for various diseases in workplaces limited by the use of expensive equipment such as spectrophotometers. Freeman et al. (2007) have demonstrated the label-less specific analysis kinase activity using a field-effect transistor device [135]. They treat sensors with ALP where sensing interface ALP can dephosphorylate enzyme kinase and then change the potential to lower level.

Advanced technologies including nanomaterials, microarrays, and lab-on-a-chip are massively exploited for ALP detection, which encourages researchers to develop better methodologies as well as design small devices for point of care applications. The following section displays recent progresses in this field.

## 8. Recent Technology

### 8.1. Optical Nanomaterials

Biological samples often have an extremely low concentration of cancer biomarkers. As a result, there is a high demand for highly ultrasensitive and selective detector probes to make it possible to detect the low levels of cancer biomarkers in biological samples. In the optical biosensors, the low levels of biomarkers are detected by using nanomaterials that help amplify the low signals by taking advantages of the nanomaterial characteristics. Table 1 illustrates number of nanomaterials that have been used so far in amplifying ALP signal in biological samples. Following the introduction of nanotechnology in the biosensor field, selectivity and sensitivity and other analytical properties of biosensors have been largely improved. Among the widely applied nanomaterials, gold nanoparticles, carbon nanotubes, photonic crystals, and graphene have stood out because of their unique ultrasensitive and selective properties [124]. For example, some of the advantages of nanomaterial properties include strong amplification effect on signals, high surface energy, superior biocompatibility, and high stability that make them excellent choices when looking for biosensor probes. Gold nanomaterials have become widely applied in the field of biosensors considering their unique advantages such as increased signal response or detection—especially for biological samples with low biomarker concentration [124]. Xia et al. (2013) also noted that gold nanoparticles improve ALP detection through signal amplification resulting in high selectivity and sensitivity [102,125,136]. Graphene nanoparticles have also been widely used in biosensors considering their unique electrical conductivity, high surface area, thermal conductivity, and electron mobility [137].

Several studies have been published using various nanostructure including nanoparticles, nanocluster, nanoflakes and carbon quantum dots alongside various optical assays to enhance ALP detection. In terms of colorimetry assays, Choi et al. (2007) used gold nanoparticles (AuNP) to increase ALP detection sensitivity [146]. The researchers used the presence of dephosphorylated Tyrosine-Arginine (Tyr-Arg) to aggregate AuNP. A model substrate for this platform was based on a phosphorylated dipeptide phospho (pTyr-Arg) with an amidated C-termini and a free N-termini for the target ALP enzyme. As such, the dephosphorylation only generated cationic Tyr-Arg. In contrast, Serizawa et al. (2010) reported the detection of ALP based on product- or substrate-dependent synthesis of AuNPs in HEPES buffer [147]. The researchers used phosphorylated dipeptide pTyr-Arg with free amidated C-termini and N-termini for the target ALP enzyme, generating cationic Tyr-Arg. The effects of enzyme and substrate concentrations on detection were explored in detail and found to be applicable to inhibitor assays. The method does not require specific storing, synthesis, or purification of AuNPs, making it a simple and facile detection technique. Li et al. (2013) developed a label-free and simple calorimetric assay to detect ALP [138]. Their sensor was based on conjugated AuNPs and adenosine triphosphate (ATP), creating a highly selective and sensitive assay. In the system, ATP induced the aggregation of cetyltrimethylammonium ammonium bromide (CTAB)-capped AuNPs while ALP stimulated disaggregation of AuNPs, thereby converting ATP into adenosine via enzymatic dephosphorylation. The presence of ALP can be visually observed (change in grey to red color) and monitored as a result of surface plasmon resonance shift and AuNP absorption band. On the other hand Song et al. (2018) proposed a facile colourimetric assay based on phosphate anion-quenched oxidase-mimicking activity of Ce(IV) ions to enhance the selective and sensitive detection of ALP activity [148]. Free Ce(IV) ions showed strong oxidase-like activity with 40 times more catalytic turnover when compared with CeO2 when catalyzing the oxidation colourless TMB into its blue product. The detection for ALP was in two linear scopes of 0–50 U/L and 50–250 U/L, and a limit down to 2.3 U/L.

In terms of SERS, Ruan et al. (2006) detected ALP activity at ultralow concentration, where AuNP was used as SERS material in 5-bromo-4-chloro-3-indolyl phosphate (BCIP) presence [139]. Enzymatic hydrolysis of BCIP contributed to the formation of indigo dyes that were noted to be highly active in detecting ALP activity at ultralow concentrations using the SERS technique. Jiang et al. further used AuNP as scattering in the presence of ALP substrate where ALP was catalysed, the ascorbic 2-phosphate (AAP) was hydrolyzed and formed ascorbic acid. The activity of the enzymatic reaction was stopped using H_3_PO_4_ and HAuCl4 was used in reacting ascorbic acid create AuNP that displayed resonance scattering (RS) with ALP product. These properties of AuNP make it possible to have a rapid electron transfer in detecting biomolecules when used as biosensors. Moreover, gold-based nanotubes also allow for rapid electron transfer and help reduce the inconsistent signal amplification of metallic nanoparticles often encountered in traditional biosensors such as low selectivity and sensitivity. Zeng et al. (2017) designed a SERS kit for ALP detection based on ’hot spots’ amplification approach [140]. The kit consisted of enzyme substrate, Ag^+^, and alkyne-tagged AuNPs for clinical assay detection of ALP in human samples. Due to electrostatic interaction, silver ions are adsorbed to the Au surface and the enzymatic catalysis of ALP trigger reduction of silver ions forming hot spots on Au core and Ag shell resulting in high SERS signal amplification. The ALP detection limit for the assay is 0.01 U/L (2.3 pg/mL) and the kit can be used at point-of-care for efficacious, reliable, and highly sensitive ALP detection potentially reducing the need for time-consuming clinical tests.

Other techniques such as naked eyes and ultraviolet–visible spectroscopy (UV-vis) have been used with different materials in detection ALP. For example, AL-Rubaee et al. (2015) used UV-vis spectroscopy in a developed platform for ALP detection in saliva-based on titanium dioxide NPs (TiO_2_ NPs) [149]. Findings from the study showed that the technique could be applied in sensitive and selective detection of ALPs in patients with gingivitis. Zhang et al. (2017) developed an inexpensive ALP detection device that uses low-cost μPADs [150]. Under an optimal environment, the new technique promotes quantitative detection of ALP in buffer samples that range from 0.075 to 5 U/mL and have a visual detection limit of 0.075 U/mL. Pandey et al. (2018) presented a platform for detecting ALP levels using -MoO_3_-x nano-flakes and zinc oxide [145]. Once ALP is introduced to the complex, there is a rapid transformation of blue -MoO_3_-x to colourless -MoO_3_ using zinc as a cofactor. The sensitivity level was found to be 0.1 M of enzyme concentration.

Fluorescence assays allow many manipulating tactics to make novel probes that take advantage of nanomaterials properties. For example, Cao et al. (2016) synthesized a fluorescence nanoprobe by combining isomers of phosphorylated fluoresceinamine on the surface of mesoporous silica-coated superparamagnetic iron oxide (Fe_3_O_4_@mSiO_2_) nanoparticle [151]. The phosphorylated fluoresceinamine was hydrolysed in the presence of ALP, leading to fluorescence recovery of ALP. Increased fluorescence intensity during high-level ALP expression provides a rapid and non-toxic method for cellular detection of ALP activity. Hu et al. (2017) proposed a new fluorescent sensing platform that uses nitrogen-doped carbon dots (N-CDs) as a probe for fluorescence signal transmission for ALP and PPi [144]. Cu^2+^ quench high fluorescent N-CDs and can be recovered when PPi is added since PPi binds to Cu^2+^. The strategy shows that N-CDs can be used in selective and sensitive detection of ALP and PPi at low detection levels of 0.16 μM and 0.4 U/L for PPi and ALP, respectively. The assay is simple, rapid, sensitive, low-cost, and convenient. More recently, nanomaterials have been used in enhancing sensitivity; for example, He and Jiao (2017) described a fluorometric technique in detecting ALP activity [142]. When ALP is added, AAP is hydrolysed, forming ascorbic acid (AA) which reduces Ag^+^ ions forming metallic silver that hinders the formation of fluorescent silver nanoclusters causing low fluorescence. There is a linear correlation in fluorescence intensity in the 1–100 U/L ALP concentration range and a detection limit of 0.63 UL. Wang et al. (2018) developed a sensitive fluorometric assay for determining ALP activity using a composite generated from copper nanoclusters (CuNCs) and flower-like cobalt oxyhydroxide (CoOOH) [143]. Upon the formation of CuNC-CoOOH aggregates, the fluorescence of CuNCs is reduced due to CoOOH sheets. If AA is added, the CoOOH sheets are reduced to Co(II) ions, recovering the fluorescence, and ALP is hydrolysed and detected at an excitation wavelength of 335 to 410 nm. The assay can result in high ALP detection to a range of 0.5 to 150 mU/mL and a detection limit of up to 0.1 mU/mL. Liu et al. (2019) used the common on-off approach to enhance sensitivity of ALP detection [141]. They functionalized gold nanoclusters by 3-aminophenylboronic acid (APBA-Au NCs). ALP can catalyze hydrolysis of phenyl phosphate to phenol, which can be subsequently hydroxylated to generate catechol in the presence of tyrosinase (TYR). Due to the special covalent combination between the catechol and boric acid group, the five-membered cyclic esters can be formed on the ligands of APBA-Au NCs—leading to the fluorescence quenching of the Au NCs.

### 8.2. Electrical Nanomaterials

Nanomaterials have also been widely applied in electrochemical biosensors as sensing probes to achieve high conductivity, catalytic activity, and biocompatibility, where they accelerate signal transduction and amplify biorecognition of biomarkers. Table 2 shows some nanomaterials used for signal amplification. Applying carbon and metal nanomaterials to modify the surface of electrodes can improve signal labelling and sensitivity, thereby enhancing the biomarker detection in complex environments. Several studies have been published where they have used nanomaterials to improve the detection of ALP. For example, Ru et al. (2013) confirmed that ionic liquid modified carbon nanotubes electrode can be applied in dynamic detection of ALP and the technique was found to be time efficient, have good precision, high sensitivity, and a wide linear range [152]. Peng et al. (2015) synthesized copper sulphide nanoparticle graphene sheets (CuS/GR) and used it in signal amplification for electrochemical detection of ALP [102,153]. The platform increased the detection of ALP in human serum linearly with the concentration of 0.1 to 100 U/L with a detection limit of 0.02 U/L. Zhou et al. (2016) created a selective and sensitive electrochemical biosensor assay for protein kinase activity [154]. Multiple signal amplification approaches using streptavidin (SiO2), SiO2 biocomposite, and carbon nanospheres (Au@C). The peptides were modified on the electrode surface after phosphorylation using protein kinase A in the presence of ATP. The ALP detection limit was 0.014 u/mL and the method showed high detection selectivity and sensitivity. Panday et al. (2017) reported a new self-aligned process for AuNP decorated polypyrrole (Ppy) biosensing microelectrode, where AuNP electro-grows out of Ppy in a selective manner [155]. The self-aligned approach promoted on-chip AuNP deposition and preparation in a single step approach saving cost and time. Zhao et al. (2018) developed a sensitive electrochemical ALP biosensor AAP as the substrate on an AuNPs decorated single-walled carbon nanotube (GNP/SWNT) and a modified glassy carbon electrode (GCE) [156]. The activity of ALP was determined indirectly according to the concentration AA, generated from AAP hydrolysis. The biosensor showed high selectivity and sensitivity at a concentration ranging 3 to 50 U/L with a detection limit of 0.2 U L-1. Simão et al. (2018) integrated CNTs within osteointegration implants and found increased ALP mineralization activity detection using the implants [157]. The device can rapidly detect ALP within blood serum, by immobilizing the covalent anti-ALP antibody towards ALP. The biosensor showed excellent performance with two linear ranges from 0.5 to 50 IU/L and from 100 to 600 IU/L and limits of detection ranged 0.25 and 84.6 IU/L, respectively. Mintz et al. (2018) created an electrochemical impedance device to detect ALP in phosphate buffer saline (PBS) solution by using anti-ALP functionalized silver nanowires (SiNWs) [127]. A three-electrode cell was used to measure electrochemical impedance where the electrode functioning as a highly disordered and very dense array of SiNWs. The device was highly efficient, selective, and sensitive in detecting very low ALP concentrations in PBS solution ranging 0.03–0.3 U/L, in addition to being highly selective to antigen and antibody interaction. The high selectivity and sensitivity make the device an effective approach for quantitative sensing and real-time detection of ALP.

### 8.3. Microarray Techniques

The microarray detection involves an advance in technology to enhance ALP detection in vitro or cell culture. Over the years, in vitro microelectrode array technology has evolved into a widely used and effective technique in studying cultured cells. Lin et al. (2008) orthogonally arranged row and column electrodes on two different glass substrates, achieving an addressable microelectrode device for the comprehensive electrochemical detection of ALP [159]. The addressable microelectrode was simple and its advantage includes ease in assembling, but it consists of only 10 × 10 addressable detection points on a single chip. The detected electrochemical response at 100 single points was achieved within 22 s. The device was used to successfully image the spots of ALP on array substrate with high-throughput imaging and detection of biochemical species. Lin et al. (2009) created a microwell array and incorporated it to the addressable device in ensuring high-throughput screening of bioparticles and genetically engineered cells [160]. The researchers demonstrated the importance of the device in rapid electrochemical detection of secreted ALP from a single genetically engineered HeLa cell using microwell/microelectrode array device. The results showed that the average decline in currency for 100 microwells is proportional to PAP concentration ranging from 0.5 to 50 μM. Murata et al. (2009) monitored the electronic expression of ALP at the single-cell level using a scanning electrochemical microscopy (SECM) [161]. The SECM measurement showed that tumor necrosis factor (TNF)-α stimulate a considerable rise in the response of transfected cells array on poly(dimethylsiloxane) (PDMS) micro-stencil without exposing them to positive-dielectrophoresis (pDEP). The overall response of untreated cells rose during the 5-h culture promoting cell adhesion and indicating that pDEP-induced stress triggers intracellular signalling, resulting in the production of ALP. Shiku et al. (2010) noted that single-cell analysis has been used as a powerful technique in constructing new types of whole cell sensors in addition to integrating individual cellular responses [162]. Nonetheless, the current sensitivity levels are insufficient when analysing a large number of data-sets since individual single-cell responses show high fluctuations. Takeda et al. (2011) microfabricated an electrochemical platform for the parallel monitoring of secreted ALP using a mammalian-cell array chip [163]. A 4 ring-ring electrode array was developed at the rim at round cellular patterns with a diameter of 270 mm. The researchers then carried out electrochemical characterization and found a collection efficiency of 50% in the dual mode when outer and inner ring electrodes were selected as the generator and collector electrodes, respectively. The present amplification mode for dual mode normal to single mode was 2.84. Ino et al. (2012) also developed a deep microwell and included them into local redox cycling–based electrochemical (LRC-EC) devices to trap single 3D culture cells and evaluate the activity of the cells [164]. Corresponding experiments and simulations were undertaken to identify the impact of deep microwells on LRC-EC system. The demonstrations that were anchored on microwell models showed that deep microwell arrays and fine interdigitated array (IDA) electrodes resulted in low cross-talk influence but high sensitivity compared to conventional techniques that use electrode arrays in comprehensive detection. The estimated time to attain a steady-state was short and this shows that the electrochemical image with 256 pixels was achieved in 10.3 s using the LRC-device. A study by Wu et al. (2016) developed a new digital single molecule electrochemical detection method (dSMED) strategy to address the problem of single molecule electrochemistry (SME) reliability based on the integration with the digital analysis in enzyme-induced metallization (EIM), which improves the application of SME in a biological system [165]. The created EIM can enhance electrochemical signals up to 100 times when compared to conventional techniques of direct oxidation of enzyme materials that offer better signal detection for single molecule detection. The incorporation of digital analysis solves the existing problems associated with fluctuations in signal detection and heterogeneity of single enzyme activities, further reducing the detection limit of ALP to 1 aM compared to the original levels of 50 aM. The use of microarrays greatly improves the reliability and accuracy of SME through the integration of a digital system that enables dSMED to ensure successful application in ALP detection in complex liver cells.

### 8.4. Lab-on-Chip Technique

Development of lab-on-chip sensors has been undertaken in advance technology to enhance ALP detection in cell culture. Lab-on-chip approaches can provide good selectivity and portable detection. Besides, the chip limits the affectance of interferences and can therefore be used in ALP detection assay in complex samples. Şen et al. (2012) examined electrochemical detection of secrete ALP secreted from single transformed HeLa cells using a chip comprising 256 microwells of single addressable sensor points, which had been modified using anti-secreted ALP [166]. The secreted ALP was successfully separated and analysed using the developed LRC-EC chip, which was found to have broad potential for single cell analysis applications. Şen et al. (2012) also proposed a new electrochemical assay for the detection of secreted ALP from transfectant HeLa cells using SECM and a microarray device [167]. The assay was reported to increase intactness and accuracy in ALP detection by eliminating undesired influence. Chen et al. (2013) developed a new and simple microfabricated device for feeder-isolated co-culture of stem cells that allows for the use of normal mouse embryonic fibroblasts (mEF) as feeder layers in addition to pure mouse embryonic stem (mES) cells without additional purification [168]. The device has significant advantages of simplicity and efficiency where the mES cells and feeder layers are spatially attached to the PDMS porous membrane and forms 3D cell colonies that have high viability. The pluripotency and self-renewal mES cells were confirmed through ALP expression. The device supported a robust and simple ALP detection in vitro stem cell co-cultures with a significant advantage in simplicity and efficiency from mEFs co-cultures without additional separation or purification. Ino et al. (2014) have recently fabricated a new type of LRC-EC chip device for the detection of a droplet array [169]. Pt pseudo-reference/counter electrodes were used to improve the electrochemical detection of redox compounds in droplets, by incorporating the individual sensors of the LRC-EC chip device. The cyclic voltammetry for LRC-EC chip with internal Pt pseudo-reference electrodes showed well-elaborate voltammograms based on redox cycling for single sensor points. The device was used in the detection of ALP activity of HeLa cells in single droplets on sensor points. The results reveal that the technology was simple, low-cost, rapid, and has the potential for clinical analysis of ALP assays. Ino et al. (2014) [170] fabricated a new LRC-EC chip device with a densely packed electrode array that has a vertical separation. The electrochemical performance of this device was assessed using amperometry and voltammetry [170]. The LRC-EC device indicated significant enhancement of electrochemical image resolution and largely applied to imaging or HRP and ALP in SG/CC and extended feedback modes. When compared to SECM imaging, electrochemical imaging, using LRC-EC systems is appropriate for continuous imaging of biomaterials since LRC-EC makes it possible to achieve rapid image acquisition. Cao et al. (2018) created a simple electrophoresis titration (ET) device for ALP detection through moving reaction boundary (MRB) [171]. In this model, ALP sped up the dephosphorylation of the 4-methylumbelliferyl phosphate disodium salt substrate in the cathode and 4-methylumbelliferone (4-MU) with blue fluorescence and negative charge under UV excitation. The 4-MU moved under the electric field into the channel and in the process resulted in the neutralization of the acidic Tris-HCl buffer, thereby creating MRB and quenching 4-MU. The sensitivity of the device was high at 0.1 U/L, and the results were better than the ones obtained from chemiluminescence, electrochemical, and colourimetric methods for ALP assays. The sensitivity of the ET technology was good (0.1 U/L), linearity (V = 0.033A + 3.87, R2 = 0.9980), stability with relative standard deviation about (2.4% to 6.8%) and recoveries (101% to 105%). The technology was successfully used to detect ALP in serum samples. Sun et al. (2019) explored a new technique for investigating a low abundance of ALP created through individual cells by the use of microfluidic droplet-based SERS method [172]. 

The researchers first created a co-flow drop-maker model in which they suspended cells in a solution using BCIP as an enzymatic substrate and AuNPs as a signal agent to encapsulate individual cells in the drops. The difference of normal liver cell lines (BNL.CL2) and hepatocellular carcinoma cell lines (HepG2) were contrasted in their ALP expression and it was observed that normal liver cells indicate that 40% lower ALP expression compared to hepatocellular carcinoma (HepG2). The results also revealed that the ALP activity of droplet-isolated cells fluctuate in a large range compared to cluster cells, even if the overall ALP expression of the two cases was similar. An ultrasensitive high output analytical process was realized for ALP detection using single cell SERS-based microfluidic droplet method.

## 9. Conclusions

This paper has thoroughly reviewed recent strategies concerning optical and electrochemical detection of ALP and has discussed the electrochemical techniques that have been addressed to make each of the techniques suitable for ALP analysis in cell culture. It has been shown that using advanced materials can enhance the signal of ALP detection, although the linear range was slightly low. Moreover, real-time detection of ALP with cells viability are monitored using microscopic images, fluorescent images and scanning electrochemical microscopy. Other effective methods includes dyes to indicate metabolic activities and the use of cell adhesion with impedance measurement. Quartz crystal microbalance, field-effect transistor and surface plasmon resonance are also advantageous methods. Real-time detection of ALP is also visible by naked eyes using advanced mobile phone cameras. Many optical and electrochemical approaches have been applied with advanced materials and recent technologies such as microarrays and lab-on-a-chip. Ino et al. have previously reviewed the bioelectrochemical applications of these technologies but focused on where analyzing and monitoring cell functions is possible [173]

In electrochemical detection strategies, the development of electrodes, electrolytes and the analysis of other parameters regarding these techniques can contribute to the development of more suitable ALP biosensors. Whereas it is obvious that a considerable amount of work needs to be performed to meet the remaining challenges, as well as limiting the influence of parameters set on electrochemical signals, these literature reviews contained in this paper suggest a strong foundation for the development of methodologies for ALP analysis. Further enhancement of the ALP detection in order to achieve the desired level of accuracy and sensitivity is required.

## Figures and Tables

**Table 1:** Number of nanomaterials for enhancing optical assays.

Detection Technique	Nanomaterials	Linear Range	Limit of Detection	Ref.
**Colorimetric**	gold nanoparticles	100–600 U/L	1000 U/L	[138]
**Raman spectroscopy**	gold nanoparticles	4 × 10^−11^ M to 4 × 10^−15^ M	4 × 10^−15^ M	[139]
**Raman spectroscopy**	gold nanoparticles	0.72 to 3 U/L	0.01 U/L	[140]
**Fluorescence**	gold nanoclusters	0.02–2.0 U/L.	0.005 U/L	[141]
**Fluorescence**	silver nanoclusters	1–100 U/L	0.63 U/L	[142]
**Fluorescence**	copper nanoclusters	0.5 to 150 mU/mL	0.1 mU/mL	[143]
**Fluorescence**	nitrogen-doped carbon dots	2.5 to 45 U/L	0.4 U/L	[144]
**Naked eye**	α-moo3- × nano-flakes	0.06 to 1 IU		[145]

**Table 2:** Number of nanomaterials for enhancing electrochemical assays.

Detection Technique	Nanomaterials	Linear Range	Limit of Detection	Ref
**Impedance**	silicon-nanowire	0.03–0.3 U/L	0.3 U/L	[127]
**Electrochemical**	copper sulfide nanoparticle	0.1 to 100 U/L	0.02 U/L	[153]
**Electrochemical**	gold nanoparticle	3 to 50 U/L	0.2 U/L	[156]
**Electrochemical**	gold nanoparticle-carbon nanotubes	0.5 to 600 IU/L	0.25 IU/L	[157]
**Photo-Electrochemical**	graphic carbon nitride (g-C_3_N_4_)/TiO_2_ nanotubes	0.3 mU/L–1 U/L	0.1 mU/L.	[158]
